# Can Interactions Between α-Synuclein, Dopamine and Calcium Explain Selective Neurodegeneration in Parkinson's Disease?

**DOI:** 10.3389/fnins.2018.00161

**Published:** 2018-03-14

**Authors:** Michael R. Post, Ori J. Lieberman, Eugene V. Mosharov

**Affiliations:** Departments of Psychiatry and Neurology, New York State Psychiatric Institute, Columbia University Medical Center, New York, NY, United States

**Keywords:** α-Synuclein, dopamine, calcium, Parkinson's disease, substantia nigra pars compacta, locus coeruleus, multiple hits

## Abstract

Several lines of evidence place alpha-synuclein (aSyn) at the center of Parkinson's disease (PD) etiology, but it is still unclear why overexpression or mutated forms of this protein affect some neuronal populations more than others. Susceptible neuronal populations in PD, dopaminergic neurons of the substantia nigra pars compacta (SNpc) and the locus coeruleus (LC), are distinguished by relatively high cytoplasmic concentrations of dopamine and calcium ions. Here we review the evidence for the multi-hit hypothesis of neurodegeneration, including recent papers that demonstrate synergistic interactions between aSyn, calcium ions and dopamine that may lead to imbalanced protein turnover and selective susceptibility of these neurons. We conclude that decreasing the levels of any one of these toxicity mediators can be beneficial for the survival of SNpc and LC neurons, providing multiple opportunities for targeted drug interventions aimed at modifying the course of PD.

## Differential susceptibility of catecholaminergic neurons in PD

Parkinson's disease (PD), the second most common neurodegenerative disorder (De Lau and Breteler, 2006), is marked by slowness (bradykinesia), resting tremor, muscular rigidity, and postural instability (Lang and Lozano, 1998). Although multiple brain regions are affected in late-stage PD (Braak et al., 1995), two catecholaminergic neuronal populations degenerate early, before the onset of the motor symptoms-dopaminergic (DA) neurons of the substantia nigra pars compacta (SNpc) and noradrenergic (NE) neurons of the locus coeruleus (LC). DA restoration through treatment with L-DOPA provides an effective symptomatic improvement, however, tolerance to treatment increases over time, accompanied by the development of severe side effects (L-DOPA-induced dyskinesia; Lewitt, 2015; Olanow, 2015). There is at the time no means available for delaying the progress of the disease, which is a critical goal in the field.

Rational design of disease-modifying therapies is complicated by the lack of a clear understanding of the pathophysiology of PD initiation and progression. The disease is predominantly sporadic, with an estimated 10% prevalence of familial cases (Eriksen et al., 2005). Alpha-synuclein (aSyn), encoded by the SNCA gene, plays a central role in both sporadic and familial PD. Mutations or multiplications of the SNCA cause autosomal dominant PD (Eriksen et al., 2005). Levels of phosphorylated aSyn are increased in post-mortem brains of PD patients and in patient-derived dopaminergic neurons (Fujiwara et al., 2002; Swirski et al., 2014). Genome-wide association studies (GWASs) have reported a correlation between the SNCA locus and the risk of developing sporadic PD (Simon-Sanchez et al., 2009; Chang et al., 2017). Importantly, post-mortem PD brains show proteinaceous aSyn-positive deposits called Lewy bodies (Spillantini et al., 1997; Baba et al., 1998). Conversely, deletion of aSyn is protective in mouse and cellular models of PD (Dauer et al., 2002; Alvarez-Fischer et al., 2008). Similarly, a recent study identified β2-adrenoreceptor (β2AR) agonists as negative regulators of the aSyn gene expression, and an association was found between the use of β2AR agonist salbutamol, a brain-penetrant asthma medication, and a reduced risk of developing PD (Mittal et al., 2017). Yet, Lewy body pathology occurs throughout the nervous system in PD patients and does not correlate well with cell death (Goedert et al., 2013; Surmeier et al., 2017a), suggesting that aSyn may be necessary but not sufficient for PD neurodegeneration.

Several cellular pathways are affected in PD, resulting in endoplasmic reticulum (ER) stress and activation of the unfolded protein response, disruption of lysosomal and proteasomal protein degradation, and impaired Ca^2+^ homeostasis and mitochondrial dysfunction (Rochet et al., 2004; Stefanis, 2012; Duda et al., 2016; Michel et al., 2016). Although there does not appear to be a unifying end-point toxicity pathway, inflammatory response and both necrotic and apoptotic degeneration are often observed in PD models (Perier et al., 2012). The central question in PD neuropathology, however, is why some neurons are highly susceptible to neurodegeneration while other, even closely related populations, are much less affected. Specifically, SNpc and LC catecholaminergic neurons degenerate in PD, whereas ventral tegmental area (VTA) and tuberoinfundibular DA neurons are relatively spared in both PD patients and laboratory models of the disorder (Hirsch et al., 1988; Braak et al., 1995). Two features of SNpc and LC neurons—the presence of elevated catecholamine and Ca^2+^ concentration in the cytosol—have consistently been suggested as modulators of their sensitivity to neurodegeneration.

Due to the ability of DA to produce oxidative stress and protein damage, it has long been speculated that a dysregulation of cytosolic DA homeostasis plays a role in PD (Edwards, 1993; Gainetdinov et al., 1998; Uhl, 1998; Schmitz et al., 2001; Lotharius and Brundin, 2002; Lohr et al., 2014; Pifl et al., 2014). Spontaneous DA oxidation at neutral pH of the cytosol yields DA-o-quinone and dopaminochrome (Graham, 1978; Sulzer and Zecca, 2000), which can then react with free cysteine and exposed cysteine residues of proteins and glutathione producing 5-S-cystenyl-DA. The latter can undergo further oxidation and is toxic to cultured cells (Spencer et al., 2002) or when injected into the mouse brain (Zhang and Dryhurst, 1994). 5-S-cystenyl adducts of DA and its metabolites are used as markers of excess cytosolic DA and oxidative stress *in vivo* (Hastings and Berman, 1999; Caudle et al., 2007) and are readily detected in human SNpc and LC, consistent with DA-induced protein damage in human PD (Fornstedt et al., 1989; Montine et al., 1995; Hastings and Berman, 1999). Other mechanisms of DA-mediated neurotoxicity include reactions of DA with nitric oxide (Daveu et al., 1997), peroxynitrite (Daveu et al., 1997; Vauzour et al., 2008) and aldehydes (Collins and Bigdeli, 1975; Deitrich and Erwin, 1980; Naoi et al., 1993; Marchitti et al., 2007). Accumulation of cytosolic DA is toxic to cells *in vitro* (Mytilineou et al., 1993; Pardo et al., 1995; Sulzer et al., 2000; Xu et al., 2002; Fuentes et al., 2007; Mosharov et al., 2009) and several reports confirm that a buildup of cytosolic DA is indeed sufficient to induce progressive nigrostriatal degeneration in rodents (Caudle et al., 2007; Chen et al., 2008), although clinical studies of L-DOPA toxicity produced controversial results (Fahn et al., 2004; Olanow et al., 2004; Holford et al., 2006).

Dysregulation of Ca^2+^ homeostasis is likewise frequently observed in models of both sporadic and familial PD (Goldberg et al., 2012; Hurley and Dexter, 2012; Surmeier et al., 2017b). This includes impairment of mitochondrial Ca^2+^ maintenance (Exner et al., 2012), disrupted communication between mitochondrial and ER Ca^2+^ stores (Ottolini et al., 2013; Guardia-Laguarta et al., 2014), decreased store-operated Ca^2+^ entry (Zhou et al., 2016), and additional mechanisms that may cause toxicity due to abnormally high or low Ca^2+^ levels (Duda et al., 2016; Michel et al., 2016; Surmeier et al., 2017b). SNpc and VTA neurons express drastically different levels of calbindin-D_28K_ (Fu et al., 2012) and those expressing high levels of this Ca^2+^ buffering protein—the majority of VTA neurons and a small percentage of SNpc neurons—are spared from neurodegeneration in PD (Yamada et al., 1990; Rcom-H'cheo-Gauthier et al., 2014). Interestingly, at least some LC neurons appear to express Ca^2+^ buffering proteins calbindin-D_28K_, calretinin and parvalbumin (Bhagwandin et al., 2013), although no comparison was made with other brain areas, such as the VTA.

SNpc neurons have long axons that extend into the striatum and arborize extensively, with many DA release sites (Matsuda et al., 2009). Physiologically, these neurons display broad action potential spikes and an autonomous tonic firing pattern governed by the activity of the L-type Ca_v_1.3 channels (LTCCs) (Hetzenauer et al., 2006; Surmeier et al., 2010). This drives a feed-forward stimulation of mitochondrial oxidative phosphorylation that maintains ATP production during increased neuronal activity (Chan et al., 2007; Surmeier et al., 2017b). Chronically increased cytoplasmic and mitochondrial Ca^2+^ levels may however drive the production of reactive oxygen and nitrogen species (ROS and RNS), leading to mitochondrial dysfunction. While Ca_v_1.3 channels are expressed at similar levels in SNpc and neighboring VTA dopaminergic neurons (Dragicevic et al., 2014), they do not drive pacemaking in VTA neurons (Chan et al., 2007; Duda et al., 2016) (although, this remains controversial Liu et al., 2014), suggesting post-translational regulation of their activity. Pharmacological blockade of LTCCs with dihydropyridines alleviates mitochondrial oxidative stress in SNpc neurons in *ex vivo* mouse brain slices (Chan et al., 2007), and protects them in neurotoxin-based models of PD (Chan et al., 2007). Similarly, LC neurons display broad action potential spikes and autonomous pacemaking that is dependent on Ca_v_1.2 and Ca_v_1.3 L-type channels (Sanchez-Padilla et al., 2014) as well as the T-type channels (Matschke et al., 2015). Dihydropyridines also prevent mitochondrial oxidative stress in LC neurons in *ex vivo* brain slices (Sanchez-Padilla et al., 2014). Although LC neurons are selectively targeted by parkinsonian neurotoxins (Masilamoni et al., 2011), the effect of LTCC blockers on the survival of LC neurons in these models has not been studied. However, an LTCC inhibitor nimodipine was shown to protect both SNpc and LC neurons in a model of chronic neuroinflammation (Hopp et al., 2015).

Overall, SNpc and LC appear to share many of the same characteristics—a proteomic analysis identified similar changes in 61 PD-associated proteins in SNpc and LC neurons (Van Dijk et al., 2012)—and are uniquely situated with high levels of cytosolic catecholamines and Ca^2+^, which in the presence of aSyn may underlie their higher susceptibility to neurodegeneration. Below, we focus on the interactions between these three chemicals, highlighting recent developments in their role toward cell-selective PD pathogenesis.

## aSyn and Ca^2+^

**Figure 1:**
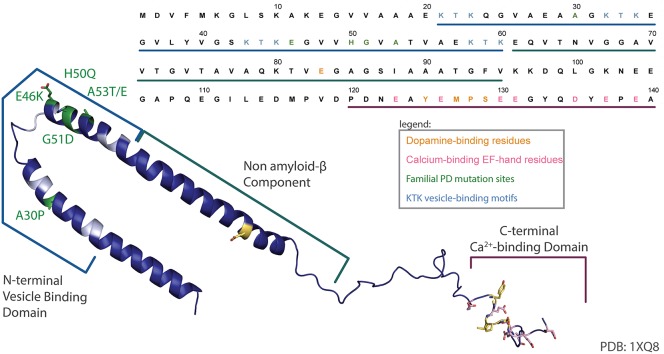
aSyn sequence map. An annotated structure of human micelle-bound aSyn solved by solution NMR (PDB 1XQ8) (Ulmer et al., 2005) and corresponding sequence have been color coded to highlight the lipid binding N-terminal domain and associated KTK motifs (cyan), common familial PD mutations (green), acidic residues in the C-terminal Ca^2+^-binding EF-hand-like motif (pink), and the dopamine-binding residues (yellow).

aSyn is a protein widely expressed in the nervous system, with a subcellular localization at the presynaptic terminal. The protein is 140 amino acids in length (Figure 1), occurs as a helically folded tetramer under physiological conditions (Bartels et al., 2011) and is able to form oligomers, fibrils and more complex aggregates, eventually leading to Lewy bodies. The N-terminus is lysine-rich and is the site of the vesicle binding, with four lipid-binding KTK motif repeats in that region. Importantly, all known SNCA familial PD mutations to date—A30P, E46K, H50Q, G51D, A53E, and A53T—are found in this domain (Rcom-H'cheo-Gauthier et al., 2014). The central region of aSyn is known as the non-amyloid-β component (NAC) of amyloid plaques found in Alzheimer's disease patients and is responsible for aSyn aggregation and Lewy body formation (Li et al., 2002). The C-terminus is comprised of an EF-hand-like sequence that is capable of binding Ca^2+^; however, overexpression of truncated aSyn that lacks the C-terminus is sufficient to elicit a PD-like phenotype in mice (Tofaris et al., 2006). Normally, aSyn is involved in regulation of synaptic vesicles exocytosis, although its exact function is still debated (Imaizumi et al., 2005; Larsen et al., 2006; Burre et al., 2010; Nemani et al., 2010; Bendor et al., 2013). Although gain-of-function mechanisms of aSyn toxicity due to its post-translational modifications or oligomerization have been widely reported, recent data suggest that the loss-of-function mechanisms may also play a role (Collier et al., 2016).

### Effects of aSyn on Ca^2+^

Intracellular Ca^2+^ is a potent second-messenger that triggers many cellular events, and its concentration is tightly regulated by the activities of transporters and channels of the plasma, ER, and mitochondrial membranes, as well as calcium-binding proteins such as calbindin, parvalbumin, and calretinin (Zaichick et al., 2017). Various mechanisms by which aSyn is able to interfere with Ca^2+^ homeostasis in different cellular compartments have been reviewed in more detail elsewhere (Duda et al., 2016; Michel et al., 2016; Ottolini et al., 2017; Surmeier et al., 2017b), but are described here briefly.

First, aSyn localizes at the mitochondria-associated membranes of the ER (MAMs) where it can regulate IP_3_ receptor-mediated transfer of calcium (Cali et al., 2012; Guardia-Laguarta et al., 2014). Pathogenic PD mutations in aSyn result in reduced association with MAM accompanied by increased mitochondrial fragmentation and augmented autophagy (Guardia-Laguarta et al., 2014). Additionally, post-translationally modified aSyn interacts with TOM20, a translocase of the outer mitochondrial membrane, and impairs mitochondrial import of proteins required for oxidative phosphorylation both *in vitro* and in postmortem brain tissue from PD patients (Di Maio et al., 2016). Second, aSyn overexpression induces lysosomal permeability, allowing lysosomal calcium and protons to leak to the cytosol and induce cell death (Bourdenx et al., 2014). Third, aSyn can increase ion permeability of the plasma membrane or interfere with the activity of its channels resulting in dysregulated neuronal firing and Ca^2+^ dynamics patterns that precede neurodegeneration (Subramaniam et al., 2014; Angelova et al., 2016). Consistently, aSyn is required for cytosolic Ca^2+^ influx through the plasma membrane following exposure to the parkinsonian neurotoxin MPP^+^ via a putative interaction with LTCCs (Lieberman et al., 2017), although the exact mechanism of this interaction needs further investigation. Fourth, a recent study showed that extracellularly added aSyn increased the activity of the Ca_v_2.2 channel, thus increasing cytoplasmic Ca^2+^ sufficiently to induce exocytotic DA release (Ronzitti et al., 2014). Interestingly, aSyn did not increase Ca_v_2.2 expression, but rather caused a relocation of Ca_v_2.2 from lipid rafts to cholesterol-poor domains, providing a novel mechanism by which aSyn may change the activity of Ca^2+^ channels via the reorganization of membrane microdomains indicating an indirect interaction between aSyn and Ca^2+^-channels. Finally, a study of the proximal aSyn intracellular partners using APEX2-based labeling found that aSyn might interact with calcineurin, a calmodulin dependent serine/threonine protein phosphatase that has ubiquitous intracellular substrates (Chung et al., 2017). This finding buttresses previous reports from the same group that demonstrated a functional relationship between aSyn levels and calcineurin activity (Caraveo et al., 2014).

### Effects of Ca^2+^ on aSyn

Ca^2+^ binding seems to promote aSyn annular oligomer formation. These ring-like oligomers have been shown to insert in the membrane forming a pore, perhaps allowing more Ca^2+^ to enter the cell (Mironov, 2015). This oligomer formation is dependent on the C-terminus and is eliminated in truncated forms of aSyn (Lowe et al., 2004). Increasing internal Ca^2+^ concentration via thapsigargin or Ca^2+^ ionophore treatment causes an increase in aggregate formation, while Ca^2+^-chelators or Ca^2+^ channel inhibitors have the opposite effect (Danzer et al., 2007; Nath et al., 2011; Follett et al., 2013). The effect of Ca^2+^ on aSyn aggregation is mediated by the Ca^2+^-activated protease, calpain, which cleaves the C-terminus of aSyn (Dufty et al., 2007; Nath et al., 2011). This has been confirmed *in vivo* by the overexpression of the calpain inhibitor, calpastatin, which reduces PD-like symptoms and pathology in a mouse model of A30P aSyn overexpression (Diepenbroek et al., 2014). Additionally, an indirect effect of Ca^2+^ on aSyn aggregation can be mediated by disruptions in autophagy. As elevated Ca^2+^ leads to increased mitochondrial stress, it has been suggested that this adds demand to proteostasis systems by necessitating increased mitochondrial turnover. This process then reduces cell's capacity to degrade aSyn, leading to aSyn aggregation (Surmeier and Schumacker, 2013).

## aSyn and DA

Due to the toxic potential of DA, it is not surprising that multiple cellular mechanisms exist to regulate its cytosolic concentration. It has been suggested that neuromelanin biosynthesis acts as one of the mechanisms for regulating toxic DA by-products by sequestering them into autophagic vacuoles (Sulzer and Zecca, 2000). Similarly, oxidized derivatives of both DA and NE are found in LC-derived neuromelanin (Wakamatsu et al., 2015). Other mechanisms include feedback inhibition of DA synthesis, catabolic DA cleavage and synaptic vesicle sequestration.

Catecholamines are synthesized from the non-essential amino acid tyrosine by a series of enzymatic reactions. In the first, rate-limiting step, tyrosine hydroxylase (TH) attaches a hydroxyl group to the aromatic ring of tyrosine, forming L-DOPA. TH activity is regulated on transcriptional, translational and post-translational levels (Goldstein and Lieberman, 1992; Kumer and Vrana, 1996; Fitzpatrick, 2000; Daubner et al., 2011), including phosphorylation-dependent activation of TH by various kinases and its inhibition by DA, which limits DA production when its cytosolic concentration increases. The second enzyme in DA biosynthesis, aromatic L-amino acid decarboxylaze (AADC), converts L-DOPA to DA. AADC activity can also be regulated by second messenger systems to decrease DA production when its extracellular concentration increases (Hadjiconstantinou and Neff, 2008). aSyn has been shown to co-immunoprecipitate with both TH (Perez et al., 2002) and AADC (Tehranian et al., 2006), and this interaction leads to decreased phosphorylation and activity of both enzymes. Decreased TH activity in the presence of aSyn overexpression depended on aSyn phosphorylation at Ser129 residue (Lou et al., 2010), which was modulated by the activity of protein phosphatase 2A (Peng et al., 2005). These data suggest that a loss of soluble aSyn due to reduced expression or aggregation may increase catecholamine synthesis.

Intracellular catecholamine catabolism starts with the cleavage by monoamine oxidase (MAO), which is localized at the outer mitochondrial membrane (Schnaitman et al., 1967), and produces two highly reactive compounds, hydrogen peroxide and 3,4-dihydroxyphenylacetaldehyde (DOPAL) (or 3,4-dihydroxyphenylglycolaldehyde for NE) (Richter, 1937). This is followed by the oxidation by aldehyde dehydrogenase (ALDH) to 3,4-dihydroxyphenylacetate (DOPAC) and 3,4-dihydroxyphenylglycol (DHPG), correspondingly. Although ALDH activity—there are both cytosolic and mitochondrial isoforms of this enzyme (Marchitti et al., 2007; Chen et al., 2014; Doorn et al., 2014) - is very high, possible neurotoxicity of the aldehyde metabolites of amines was predicted 60 years ago due to their extremely reactive nature (Blaschko, 1952). Indeed, the presence of DOPAL and its metabolites has been demonstrated both *in vitro* and *in vivo* (Burke et al., 2004; Goldstein et al., 2014). Additionally, a line of mice that are deficient for ALDH1a1 and ALDH2, the cytosolic and the mitochondrial isoforms expressed in SNpc DA neurons (Mccaffery and Drager, 1994; Galter et al., 2003), showed age-dependent, L-DOPA-responsive deficits in motor performance, significant increases in biogenic aldehydes and a loss of SNpc DA neurons (Wey et al., 2012), confirming that impaired detoxification of biogenic aldehydes may cause PD-like degeneration.

Finally, sequestration and compartmentalization of DA inside secretory vesicles is achieved via the activity of vesicular monoamine transporters (VMAT). The enzyme responsible for the conversion of DA to NE in noradrenergic neurons, dopamine beta-hydroxylase, is located in the lumen and the membrane of synaptic vesicles. Moreover, the acidic pH of vesicles prevents auto-oxidation of DA and NE, allowing high vesicular neurotransmitter concentrations without the formation of reactive species. Importantly, synaptic vesicle membrane is “leaky” and *in vitro* and *in vivo* studies have demonstrated that leakage of catecholamines from storage vesicles is the primary source of their catabolism in the cytosol (Goldstein et al., 1988; Halbrugge et al., 1989; Tyce et al., 1995).

High cytosolic DA levels following L-DOPA treatment have been shown to induce selective SNpc neuron degeneration and the formation of neuromelanin (Sulzer et al., 2000), whereas increased loading of DA from cytosol to vesicles following overexpression of vesicular monoamine transporter 2 (VMAT2) provides neuroprotection from L-DOPA (Mosharov et al., 2009). Consistent with this, higher levels of neuromelanin are found in SNpc neurons that degenerate in PD (Zucca et al., 2014). Striatal DA synaptic vesicles from PD patients were also found to have lower levels of VMAT2 (Pifl et al., 2014), although as these patients were almost certainly treated with L-DOPA, a decrease in VMAT expression could be a compensatory response rather than a cause of PD.

Oxidized DA and other catecholamines are able to interact with aSyn, producing DA-modified aSyn, which is less likely to fibrilize and instead forms soluble oligomers (Conway et al., 2001; Rochet et al., 2004). This interaction is non-covalent, reversible and occurs at the Y_125_EMPS_129_ pentapeptide in the C-terminal region of α-Syn with an additional long-range electrostatic interaction with E83 in the nAC region (Figure 1, in yellow) (Mazzulli et al., 2007; Herrera et al., 2008). Using fluorescence-lifetime imaging microscopy to monitor the relative position of the N- and C- terminals of aSyn, it was shown that DA induces a conformation where the termini are closer together, which may inhibit fibril formation (Outeiro et al., 2009). Additionally, DOPAL may cross-link aSyn lysine residues, also facilitating its aggregation (Werner-Allen et al., 2016). Intracellular aSyn oligomeric species can be cytotoxic by a variety of mechanisms, including permeabilization of vesicular and plasma membranes by pore-forming fibrils (Ding et al., 2002; Gosavi et al., 2002; Lashuel et al., 2002; Mosharov et al., 2006), disruption of proteasomal protein clearance, chronic ER stress, mitochondrial dysfunction and inhibition of SNARE complex formation and neurotransmitter release (Rochet et al., 2004; Ebrahimi-Fakhari et al., 2011; Choi et al., 2013; Kalia et al., 2013; Zaltieri et al., 2015).

Monomeric DA-aSyn, however, may also be toxic by interfering with protein degradation via a lysosomal pathway called chaperone-mediated autophagy (CMA) (Cuervo et al., 2004, 2010). CMA cytosolic substrates contain a KFERQ-like motif that can be recognized by the chaperone protein cyt-Hsc70 that delivers them to a lysosomal associated membrane protein (LAMP2A). LAMP2A forms a translocation complex once bound to a substrate and the unfolded protein crosses into the lysosomal lumen where it can be degraded. While aSyn, oxidized aSyn, and a phosphomimetic S129E aSyn mutant show similar LAMP2A binding levels, lysosomal uptake of the latter is significantly diminished. DA-aSyn demonstrates a similar CMA profile when compared to phosphorylated aSyn in that it binds to the lysosome without evidence of translocation. Furthermore, unlike phosphorylated aSyn, DA-aSyn blocks both the binding and uptake of a CMA substrate GAPDH, suggesting stronger binding to LAMP2A. A mutation in the DA-interacting region of aSyn (Y_125_EMPS_129_ to F_125_AAFA_129_) nullifies the effect, further demonstrating that the interaction of DA and oxidized forms of DA with aSyn leads to this change in CMA. In primary neuronal cultures, the same CMA blockade was demonstrated after exposure to a high dose of L-DOPA, but not in neurons derived from aSyn null animals (Martinez-Vicente et al., 2008).

A hypothesis that decreased uptake of DA into synaptic vesicles should lead to PD-like nigrostriatal neurodegeneration due to increased cytosolic transmitter levels was examined in mice that displayed a 95% reduction of VMAT2 expression due to a hypomorphic allele (Caudle et al., 2007). Surprisingly, the first generation of these mice (VMAT2-deficient KA1 line Mooslehner et al., 2001) did not show any PD phenotype, despite an ~85% reduction in brain levels of DA, NE and serotonin and their increased turnover. It was subsequently discovered, however, that this mouse line had a spontaneous deletion of the SNCA gene (Specht and Schoepfer, 2001; Colebrooke et al., 2006). After further breeding to reintroduce the wild-type aSyn gene, the resulting VMAT2-LO mice showed signs of PD-like progressive neurodegeneration, including L-DOPA-responsive motor deficits, oxidative stress and protein damage, decreased DA, DAT, and TH levels in the striatum, and pathological accumulations of aSyn and a reduced number of DA neurons in the SNpc (Caudle et al., 2007; Taylor et al., 2011). Overall, the VMAT2-LO mouse model not only demonstrated that a reduced capacity of cells to sequester cytosolic DA is sufficient to cause PD-like degeneration of neurons and their axonal projections, but also that this effect requires the presence of aSyn.

Another recent study investigated the toxic interaction between aSyn and DA *in vivo* by combining a common familial PD aSyn mutation with elevated cytosolic DA (Mor et al., 2017). Mice that overexpress PD mutant A53T aSyn were injected with a lentivirus containing TH with an R_37_R_38_ to E_37_E_38_ mutation. This mutation leads to a loss of feedback inhibition of TH by DA, resulting in increased neurotransmitter production in the cytosol (Nakashima et al., 2002). Elevation of cellular DA levels induced progressive motor impairment accompanied by nigrostriatal degeneration and increased formation of aSyn oligomers in A53T aSyn overexpressing mice but not in WT. Furthermore, in *Caenorhabditis elegans* overexpressing A53T aSyn, DA toxicity was prevented if DA-interacting residues of aSyn were mutated (Mor et al., 2017). Overall, both *in vitro* and *in vivo* data suggest that DA and aSyn have a synergetic effect on toxicity and that decreasing the levels of either of the compounds is neuroprotective.

## aSyn, DA and Ca^2+^

Ca^2+^ levels positively regulate the activity of both TH and AADC, providing a direct connection between synaptic activity and DA synthesis. However, because of Ca^2+^-driven pacemaking in SNpc and LC neurons, elevated levels of Ca^2+^ also lead to chronically increased cytosolic catecholamine levels. In agreement with this, L-DOPA treatment produces higher concentration of cytosolic catecholamines in cultured SNpc (Mosharov et al., 2009) and LC (unpublished data) compared to VTA neurons, which translated into higher susceptibility of these neurons to L-DOPA-induced degeneration. The difference between these cell types was normalized by pharmacological or genetic blockade of the LTCCs, confirming their role in selective PD-like neurodegeneration. Importantly, deletion of aSyn also protected SNpc neurons from L-DOPA-induced toxicity without changing cytosolic DA concentration, demonstrated that the levels of Ca^2+^, DA and aSyn are equally important for toxicity.

Using the same model system, we recently investigated metabolic changes in neurons exposed to the parkinsonian neurotoxin MPP^+^ (Lieberman et al., 2017). Similar to the difference observed *in vivo* described above, a significantly higher level of toxicity was observed in cultured SNpc than VTA neurons. In MPP^+^-treated SNpc, but not VTA neurons, neurotoxicity was caused by a transient increase in cytosolic Ca^2+^ that required the activity of LTCCs and ryanodine receptors. Combined with MPP^+^-mediated inhibition of DA cleavage by MAO (Choi et al., 2015), this caused upregulation of cytosolic DA and nitric oxide levels, mitochondria oxidation, and ER stress. As with L-DOPA toxicity, SNpc neurons from aSyn deficient mice were significantly more resistant to MPP^+^. Thus, in two different toxicity models we found that selective death of SNpc neurons results from a combination of “multiple hits,” including the activity of the LTCCs that create high basal cytoplasmic Ca^2+^ levels, an upregulation of DA synthesis and the presence of aSyn. Similar upregulation of Ca^2+^/NO with concomitant mitochondria oxidative stress was demonstrated in LC neurons (Sanchez-Padilla et al., 2014) and SN neurons exposed to preformed aSyn fibrils (Dryanovski et al., 2013), indicating that this pathway may be commonly activated under stress conditions.

A recent study of DA- and aSyn-mediated toxicity in human idiopathic and familial iPSC-derived DA neurons from patients with a DJ-1 mutation (PARK7) provided more evidence for the involvement of multiple factors in mediating PD-like neurotoxicity (Burbulla et al., 2017). The authors identified a DA- and Ca^2+^-dependent toxic cascade that started with mitochondrial oxidative stress leading to lysosomal dysfunction and aSyn accumulation. Interestingly, this toxicity pathway was not present in DJ-1 deficient mice or mouse iPSC-derived DA neurons generated from DJ-1 KO fibroblasts unless either DA production or aSyn expression was increased. Underlying species-specific differences may therefore explain the difficulties of creating an appropriate mouse model of PD.

## Concluding remarks and future directions

**Figure 2:**
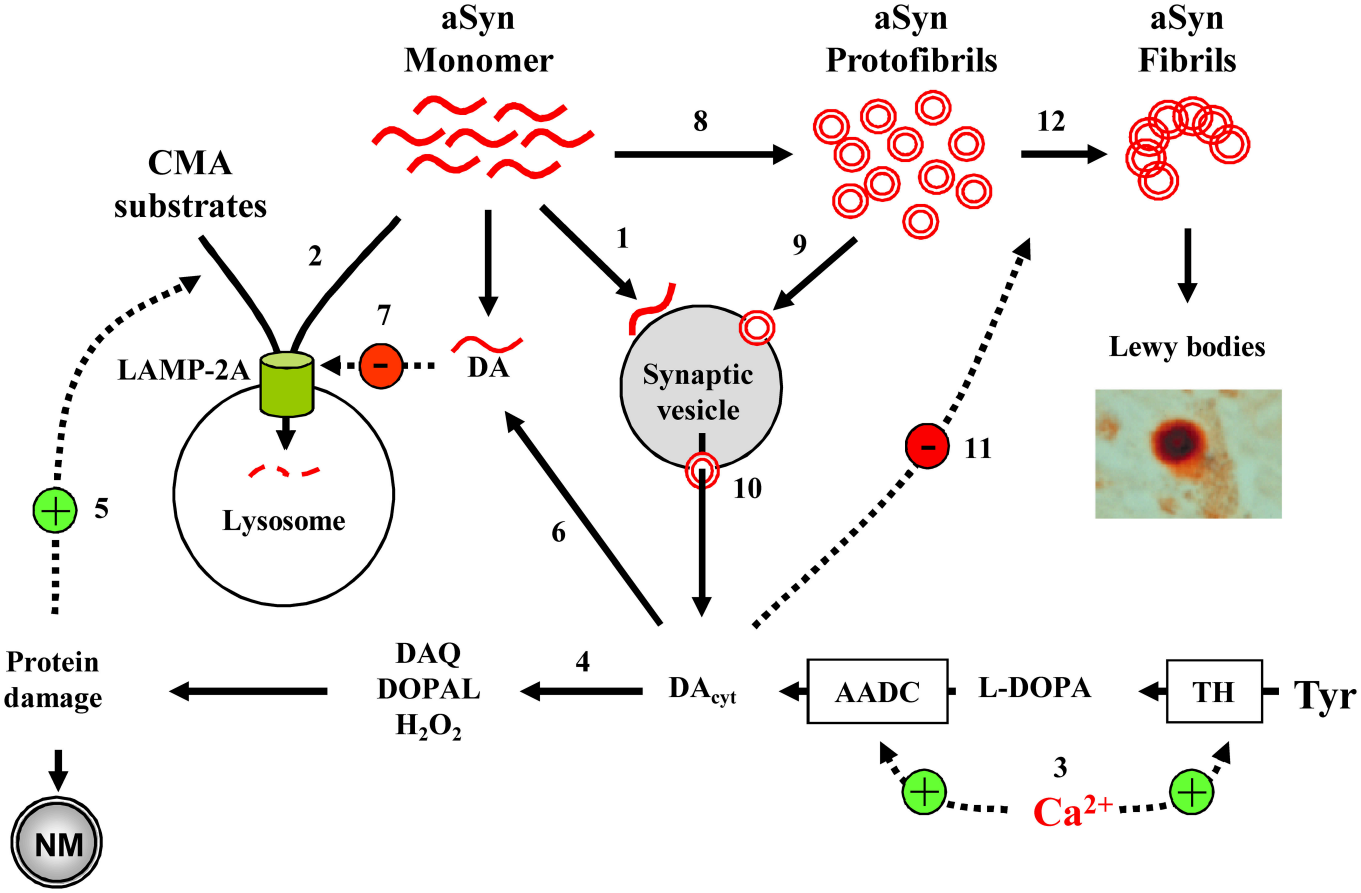
Model of cell-selective PD pathogenesis. **(1)** A physiological function of aSyn may be to bind synaptic vesicles in a reversible manner to inhibit exocytosis. **(2)** aSyn is degraded by CMA following LAMP-2A-mediated transport into the lysosomes. **(3)** In SN and LC neurons, high Ca^2+^ levels upregulate TH and AADC, leading to increased DA concentrations and **(4)** associated oxyradical stress, which induces various cell defense mechanisms, including **(5)** CMA. **(6)** DA-modified aSyn **(7)** blocks LAMP-2A-mediated uptake of CMA substrates, including aSyn itself. **(8)** aSyn may oligomerize to toxic protofibrils, which can **(9)** bind to and **(10)** permeabilize synaptic vesicles, leading to further increase in cytosolic DA. **(11)** DA stabilizes aSyn protofibrils, inhibiting the formation of larger polymers of aSyn **(12)**. Overall, the presence of such interactions where DA and aSyn act as independent stressors that converge to produce neurotoxicity may explain why catecholaminergic neurons with Ca^+2^ channel- mediated pacemaking are more prone to produce neuromelanin and Lewy bodies, and are particularly vulnerable in PD and parkinsonian animal models. Only a few aSyn-DA-Ca^2+^ interactions discussed in the text are shown.

At the center of PD pathology is aSyn, which tends to form soluble oligomers and insoluble fibrils. Oligomerization is increased with increased Ca^2+^ or DA levels, while aSyn oligomers are able to increase internal Ca^2+^ and DA concentrations, forming a potential positive feedback cycle. Furthermore, DA-modified aSyn blocks CMA-mediated protein degradation, potentially causing a buildup of monomeric aSyn that then aggregates into more oligomers (Figure 2). These interactions demonstrate the precarious nature of SNpc and LC neuron health as, if one aspect of the homeostatic processes goes awry, the feedback loops activate and neurotoxicity ensues. Importantly, in this model it is possible to initiate the pathological sequence of events that lead to neurodegeneration by diverse insults, including elevation of Ca^2+^ levels, increased cytosolic DA unrelated to Ca^2+^-dependent regulation, mutation or overexpression of α-Syn, inhibition of CMA activity due to aging (Schneider et al., 2014, 2015), the presence of other parkinsonian mutations or other possible mechanisms.

Therapeutically, this hypothesis provides several avenues to pursue the disease-modifying opportunities as decreasing the levels of any one of these key toxicity mediators should be beneficial for the survival of SNpc and LC neurons. Previous work has demonstrated the utility of immunotherapy to reduce aSyn levels in the CNS (Masliah et al., 2005) and prevent possible trans-synaptic spread of toxic aSyn species (Bae et al., 2012). A retrospective analysis demonstrated that the use of dihydropyridines correlates with decreased probability of developing PD (Pasternak et al., 2012), and an LTCC antagonist isradipine is currently in phase III clinical trials as a disease-modifying therapy for PD (Swart and Hurley, 2016). Combining these approaches with drugs that reduce toxic DA species might provide additional benefits. One important future focus will be the development of diagnostic tools to enable earlier disease-modifying treatments and stratification of patient populations to enhance beneficial outcomes. The level of aSyn peripherally and in the CNS (Malek et al., 2014) as well as the status of DA homeostasis (Niethammer et al., 2012) are currently the focus of studies aimed at developing bioassays and imaging approaches to identify pre-symptomatic PD cases with defined patho-physiologies to give “personalized” treatments.

## Author contributions

MP, OL, and EM gave their substantial contribution to conception and design of the manuscript, its drafting and revising it critically. All authors have approved the manuscript in its present form for publication and agree to be accountable for all aspects of the work.

### Conflict of interest statement

The authors declare that the research was conducted in the absence of any commercial or financial relationships that could be construed as a potential conflict of interest.

## References

[B1] Alvarez-Fischer D. Henze C. Strenzke C. Westrich J. Ferger B. Hoglinger G. U.. (2008). Characterization of the striatal 6-OHDA model of Parkinson's disease in wild type and alpha-synuclein-deleted mice. Exp. Neurol. 210, 182–193. 10.1016/j.expneurol.2007.10.012 18053987

[B2] Angelova P. R. Ludtmann M. H. Horrocks M. H. Negoda A. Cremades N. Klenerman D.. (2016). Ca^2+^ is a key factor in alpha-synuclein-induced neurotoxicity. J. Cell Sci. 129, 1792–1801. 10.1242/jcs.180737 26989132 PMC4893653

[B3] Baba M. Nakajo S. Tu P. H. Tomita T. Nakaya K. Lee V. M.. (1998). Aggregation of alpha-synuclein in Lewy bodies of sporadic Parkinson's disease and dementia with Lewy bodies. Am. J. Pathol. 152, 879–884. 9546347 PMC1858234

[B4] Bae E. J. Lee H. J. Rockenstein E. Ho D. H. Park E. B. Yang N. Y.. (2012). Antibody-aided clearance of extracellular alpha-synuclein prevents cell-to-cell aggregate transmission. J. Neurosci. 32, 13454–13469. 10.1523/JNEUROSCI.1292-12.2012 23015436 PMC3752153

[B5] Bartels T. Choi J. G. Selkoe D. J. (2011). alpha-Synuclein occurs physiologically as a helically folded tetramer that resists aggregation. Nature 477, 107–110. 10.1038/nature10324 21841800 PMC3166366

[B6] Bendor J. T. Logan T. P. Edwards R. H. (2013). The function of alpha-synuclein. Neuron 79, 1044–1066. 10.1016/j.neuron.2013.09.004 24050397 PMC3866954

[B7] Bhagwandin A. Gravett N. Bennett N. C. Manger P. R. (2013). Distribution of parvalbumin, calbindin and calretinin containing neurons and terminal networks in relation to sleep associated nuclei in the brain of the giant Zambian mole-rat (*Fukomys mechowii*). J. Chem. Neuroanat. 52, 69–79. 10.1016/j.jchemneu.2013.06.002 23796985

[B8] Blaschko H. (1952). Amine oxidase and amine metabolism. Pharmacol. Rev. 4, 415–458. 13026407

[B9] Bourdenx M. Bezard E. Dehay B. (2014). Lysosomes and alpha-synuclein form a dangerous duet leading to neuronal cell death. Front. Neuroanat. 8:83. 10.3389/fnana.2014.00083 25177278 PMC4132369

[B10] Braak H. Braak E. Yilmazer D. Schultz C. de Vos R. A. Jansen E. N. (1995). Nigral and extranigral pathology in Parkinson's disease. J. Neural Transm. (Suppl. 46), 15–31. 8821039

[B11] Burbulla L. F. Song P. Mazzulli J. R. Zampese E. Wong Y. C. Jeon S.. (2017). Dopamine oxidation mediates mitochondrial and lysosomal dysfunction in Parkinson's disease. Science 357, 1255–1261. 10.1126/science.aam9080 28882997 PMC6021018

[B12] Burke W. J. Li S. W. Chung H. D. Ruggiero D. A. Kristal B. S. Johnson E. M.. (2004). Neurotoxicity of MAO metabolites of catecholamine neurotransmitters: role in neurodegenerative diseases. Neurotoxicology 25, 101–115. 10.1016/S0161-813X(03)00090-1 14697885

[B13] Burre J. Sharma M. Tsetsenis T. Buchman V. Etherton M. R. Sudhof T. C. (2010). Alpha-synuclein promotes SNARE-complex assembly *in vivo* and *in vitro*. Science 329, 1663–1667. 10.1126/science.1195227 20798282 PMC3235365

[B14] Cali T. Ottolini D. Negro A. Brini M. (2012). alpha-synuclein controls mitochondrial calcium homeostasis by enhancing endoplasmic reticulum-mitochondria interactions. J. Biol. Chem. 287, 17914–17929. 10.1074/jbc.M111.302794 22453917 PMC3365710

[B15] Caraveo G. Auluck P. K. Whitesell L. Chung C. Y. Baru V. Mosharov E. V.. (2014). Calcineurin determines toxic versus beneficial responses to alpha-synuclein. Proc. Natl. Acad. Sci. U.S.A. 111, E3544–E3552. 10.1073/pnas.1413201111 25122673 PMC4151770

[B16] Caudle W. M. Richardson J. R. Wang M. Z. Taylor T. N. Guillot T. S. Mccormack A. L. (2007). Reduced vesicular storage of dopamine causes progressive nigrostriatal neurodegeneration. J. Neurosci. 27, 8138–8148. 10.1523/JNEUROSCI.0319-07.2007 17652604 PMC6672727

[B17] Chan C. S. Guzman J. N. Ilijic E. Mercer J. N. Rick C. Tkatch T.. (2007). ‘Rejuvenation’ protects neurons in mouse models of Parkinson's disease. Nature 447, 1081–1086. 10.1038/nature05865 17558391

[B18] Chang D. Nalls M. A. Hallgrimsdottir I. B. Hunkapiller J. Van Der Brug M. Cai F.. (2017). A meta-analysis of genome-wide association studies identifies 17 new Parkinson's disease risk loci. Nat. Genet. 49, 1511–1516. 10.1038/ng.3955 28892059 PMC5812477

[B19] Chen C. H. Ferreira J. C. Gross E. R. Mochly-Rosen D. (2014). Targeting aldehyde dehydrogenase 2: new therapeutic opportunities. Physiol. Rev. 94, 1–34. 10.1152/physrev.00017.2013 24382882 PMC3929114

[B20] Chen L. Ding Y. Cagniard B. Van Laar A. D. Mortimer A. Chi W.. (2008). Unregulated cytosolic dopamine causes neurodegeneration associated with oxidative stress in mice. J. Neurosci. 28, 425–433. 10.1523/JNEUROSCI.3602-07.2008 18184785 PMC6670521

[B21] Choi B. K. Choi M. G. Kim J. Y. Yang Y. Lai Y. Kweon D. H.. (2013). Large alpha-synuclein oligomers inhibit neuronal SNARE-mediated vesicle docking. Proc. Natl. Acad. Sci. U.S.A. 110, 4087–4092. 10.1073/pnas.1218424110 23431141 PMC3593925

[B22] Choi S. J. Panhelainen A. Schmitz Y. Larsen K. E. Kanter E. Wu M.. (2015). Changes in neuronal dopamine homeostasis following 1-methyl-4-phenylpyridinium (MPP+) exposure. J. Biol. Chem. 290, 6799–6809. 10.1074/jbc.M114.631556 25596531 PMC4358106

[B23] Chung C. Y. Khurana V. Yi S. Sahni N. Loh K. H. Auluck P. K.. (2017). *In situ* peroxidase labeling and mass-spectrometry connects alpha-synuclein directly to endocytic trafficking and mRNA metabolism in neurons. Cell Syst. 4, 242–250. 10.1016/j.cels.2017.01.002 28131823 PMC5578869

[B24] Colebrooke R. E. Humby T. Lynch P. J. Mcgowan D. P. Xia J. Emson P. C. (2006). Age-related decline in striatal dopamine content and motor performance occurs in the absence of nigral cell loss in a genetic mouse model of Parkinson's disease. Eur. J. Neurosci. 24, 2622–2630. 10.1111/j.1460-9568.2006.05143.x 17100850

[B25] Collier T. J. Redmond D. E. Jr. Steece-Collier K. Lipton J. W. Manfredsson F. P. (2016). Is alpha-synuclein loss-of-function a contributor to Parkinsonian pathology? Evidence from non-human primates. Front. Neurosci. 10:12. 10.3389/fnins.2016.00012 26858591 PMC4731516

[B26] Collins M. A. Bigdeli M. G. (1975). Tetrahydroisoquinolines *in vivo*. I. Rat brain formation of salsolinol, a condensation product of dopamine and acetaldehyde, under certain conditions during ethanol intoxication. Life Sci. 16, 585–601. 1168298

[B27] Conway K. A. Rochet J. C. Bieganski R. M. Lansbury P. T. Jr. (2001). Kinetic stabilization of the alpha-synuclein protofibril by a dopamine-alpha-synuclein adduct. Science 294, 1346–1349. 10.1126/science.1063522 11701929

[B28] Cuervo A. M. Stefanis L. Fredenburg R. Lansbury Pt J. Sulzer D. (2004). Impaired degradation of mutant alpha-synuclein by chaperone-mediated autophagy. Science 305, 1292–1295. 10.1126/science.1101738 15333840

[B29] Cuervo A. M. Wong E. S. Martinez-Vicente M. (2010). Protein degradation, aggregation, and misfolding. Mov. Disord. 25(Suppl. 1), S49–S54. 10.1002/mds.22718 20187257

[B30] Danzer K. M. Haasen D. Karow A. R. Moussaud S. Habeck M. Giese A.. (2007). Different species of alpha-synuclein oligomers induce calcium influx and seeding. J. Neurosci. 27, 9220–9232. 10.1523/JNEUROSCI.2617-07.2007 17715357 PMC6672196

[B31] Daubner S. C. Le T. Wang S. (2011). Tyrosine hydroxylase and regulation of dopamine synthesis. Arch. Biochem. Biophys. 508, 1–12. 10.1016/j.abb.2010.12.017 21176768 PMC3065393

[B32] Dauer W. Kholodilov N. Vila M. Trillat A. C. Goodchild R. Larsen K. E.. (2002). Resistance of alpha -synuclein null mice to the parkinsonian neurotoxin MPTP. Proc. Natl. Acad. Sci. U.S.A. 99, 14524–14529. 10.1073/pnas.172514599 12376616 PMC137916

[B33] Daveu C. Servy C. Dendane M. Marin P. Ducrocq C. (1997). Oxidation and nitration of catecholamines by nitrogen oxides derived from nitric oxide. Nitric Oxide 1, 234–243. 10.1006/niox.1997.0123 9704585

[B34] Deitrich R. Erwin V. (1980). Biogenic amine-aldehyde condensation products: tetrahydroisoquinolines and tryptolines (beta-carbolines). Annu. Rev. Pharmacol. Toxicol. 20, 55–80. 10.1146/annurev.pa.20.040180.000415 6992705

[B35] De Lau L. M. Breteler M. M. (2006). Epidemiology of Parkinson's disease. Lancet Neurol. 5, 525–535. 10.1016/S1474-4422(06)70471-9 16713924

[B36] Diepenbroek M. Casadei N. Esmer H. Saido T. C. Takano J. Kahle P. J. (2014). Overexpression of the calpain-specific inhibitor calpastatin reduces human alpha-Synuclein processing, aggregation and synaptic impairment in [A30P]alphaSyn transgenic mice. Hum. Mol. Genet. 23, 3975–3989. 10.1093/hmg/ddu112 24619358 PMC4110482

[B37] Di Maio R. Barrett P. J. Hoffman E. K. Barrett C. W. Zharikov A. Borah A.. (2016). alpha-Synuclein binds to TOM20 and inhibits mitochondrial protein import in Parkinson's disease. Sci. Transl. Med. 8:342ra378. 10.1126/scitranslmed.aaf3634 27280685 PMC5016095

[B38] Ding T. T. Lee S. J. Rochet J. C. Lansbury P. T. Jr. (2002). Annular alpha-synuclein protofibrils are produced when spherical protofibrils are incubated in solution or bound to brain-derived membranes. Biochemistry 41, 10209–10217. 10.1021/bi020139h 12162735

[B39] Doorn J. A. Florang V. R. Schamp J. H. Vanle B. C. (2014). Aldehyde dehydrogenase inhibition generates a reactive dopamine metabolite autotoxic to dopamine neurons. Parkinsonism Relat Disord 20(Suppl. 1), S73–S75. 10.1016/S1353-8020(13)70019-1 24262193 PMC3932615

[B40] Dragicevic E. Poetschke C. Duda J. Schlaudraff F. Lammel S. Schiemann J.. (2014). Cav1.3 channels control D2-autoreceptor responses via NCS-1 in substantia nigra dopamine neurons. Brain 137, 2287–2302. 10.1093/brain/awu131 24934288 PMC4107734

[B41] Dryanovski D. I. Guzman J. N. Xie Z. Galteri D. J. Volpicelli-Daley L. A. Lee V. M.. (2013). Calcium entry and alpha-synuclein inclusions elevate dendritic mitochondrial oxidant stress in dopaminergic neurons. J. Neurosci. 33, 10154–10164. 10.1523/JNEUROSCI.5311-12.2013 23761910 PMC3682382

[B42] Duda J. Potschke C. Liss B. (2016). Converging roles of ion channels, calcium, metabolic stress, and activity pattern of Substantia nigra dopaminergic neurons in health and Parkinson's disease. J. Neurochem. 139(Suppl. 1), 156–178. 10.1111/jnc.13572 26865375 PMC5095868

[B43] Dufty B. M. Warner L. R. Hou S. T. Jiang S. X. Gomez-Isla T. Leenhouts K. M.. (2007). Calpain-cleavage of alpha-synuclein: connecting proteolytic processing to disease-linked aggregation. Am. J. Pathol. 170, 1725–1738. 10.2353/ajpath.2007.061232 17456777 PMC1854966

[B44] Ebrahimi-Fakhari D. Cantuti-Castelvetri I. Fan Z. Rockenstein E. Masliah E. Hyman B. T.. (2011). Distinct roles *in vivo* for the ubiquitin-proteasome system and the autophagy-lysosomal pathway in the degradation of alpha-synuclein. J. Neurosci. 31, 14508–14520. 10.1523/JNEUROSCI.1560-11.2011 21994367 PMC3587176

[B45] Edwards R. H. (1993). Neural degeneration and the transport of neurotransmitters. Ann. Neurol. 34, 638–645. 10.1002/ana.410340504 7902065

[B46] Eriksen J. L. Przedborski S. Petrucelli L. (2005). Gene dosage and pathogenesis of Parkinson's disease. Trends Mol. Med. 11, 91–96. 10.1016/j.molmed.2005.01.001 15760766

[B47] Exner N. Lutz A. K. Haass C. Winklhofer K. F. (2012). Mitochondrial dysfunction in Parkinson's disease: molecular mechanisms and pathophysiological consequences. EMBO J. 31, 3038–3062. 10.1038/emboj.2012.170 22735187 PMC3400019

[B48] Fahn S. Oakes D. Shoulson I. Kieburtz K. Rudolph A. Lang A.. (2004). Levodopa and the progression of Parkinson's disease. N. Engl. J. Med. 351, 2498–2508. 10.1056/NEJMoa033447 15590952

[B49] Fitzpatrick P. F. (2000). The aromatic amino acid hydroxylases. Adv. Enzymol. Relat. Areas Mol. Biol. 74, 235–294. 10.1002/9780470123201.ch6 10800597

[B50] Follett J. Darlow B. Wong M. B. Goodwin J. Pountney D. L. (2013). Potassium depolarization and raised calcium induces alpha-synuclein aggregates. Neurotox. Res. 23, 378–392. 10.1007/s12640-012-9366-z 23250862

[B51] Fornstedt B. Brun A. Rosengren E. Carlsson A. (1989). The apparent autoxidation rate of catechols in dopamine-rich regions of human brains increases with the degree of depigmentation of substantia nigra. J. Neural Transm. Park. Dis. Dement. Sect. 1, 279–295. 10.1007/BF02263482 2597314

[B52] Fu Y. Yuan Y. Halliday G. Rusznak Z. Watson C. Paxinos G. (2012). A cytoarchitectonic and chemoarchitectonic analysis of the dopamine cell groups in the substantia nigra, ventral tegmental area, and retrorubral field in the mouse. Brain Struct. Funct. 217, 591–612. 10.1007/s00429-011-0349-2 21935672

[B53] Fuentes P. Paris I. Nassif M. Caviedes P. Segura-Aguilar J. (2007). Inhibition of VMAT-2 and DT-diaphorase induce cell death in a substantia nigra-derived cell line–an experimental cell model for dopamine toxicity studies. Chem. Res. Toxicol. 20, 776–783. 10.1021/tx600325u 17425337

[B54] Fujiwara H. Hasegawa M. Dohmae N. Kawashima A. Masliah E. Goldberg M. S.. (2002). alpha-Synuclein is phosphorylated in synucleinopathy lesions. Nat. Cell Biol. 4, 160–164. 10.1038/ncb748 11813001

[B55] Gainetdinov R. R. Fumagalli F. Wang Y. M. Jones S. R. Levey A. I. Miller G. W.. (1998). Increased MPTP neurotoxicity in vesicular monoamine transporter 2 heterozygote knockout mice. J. Neurochem. 70, 1973–1978. 10.1046/j.1471-4159.1998.70051973.x 9572281

[B56] Galter D. Buervenich S. Carmine A. Anvret M. Olson L. (2003). ALDH1 mRNA: presence in human dopamine neurons and decreases in substantia nigra in Parkinson's disease and in the ventral tegmental area in schizophrenia. Neurobiol. Dis. 14, 637–647. 10.1016/j.nbd.2003.09.001 14678778

[B57] Goedert M. Spillantini M. G. Del Tredici K. Braak H. (2013). 100 years of Lewy pathology. Nat. Rev. Neurol. 9, 13–24. 10.1038/nrneurol.2012.242 23183883

[B58] Goldberg J. A. Guzman J. N. Estep C. M. Ilijic E. Kondapalli J. Sanchez-Padilla J.. (2012). Calcium entry induces mitochondrial oxidant stress in vagal neurons at risk in Parkinson's disease. Nat. Neurosci. 15, 1414–1421. 10.1038/nn.3209 22941107 PMC3461271

[B59] Goldstein D. S. Eisenhofer G. Stull R. Folio C. J. Keiser H. R. Kopin I. J. (1988). Plasma dihydroxyphenylglycol and the intraneuronal disposition of norepinephrine in humans. J. Clin. Invest. 81, 213–220. 10.1172/JCI113298 3335637 PMC442496

[B60] Goldstein D. S. Kopin I. J. Sharabi Y. (2014). Catecholamine autotoxicity. Implications for pharmacology and therapeutics of Parkinson disease and related disorders. Pharmacol Ther. 144, 268–282. 10.1016/j.pharmthera.2014.06.006 24945828 PMC4591072

[B61] Goldstein M. Lieberman A. (1992). The role of the regulatory enzymes of catecholamine synthesis in Parkinson's disease. Neurology 42, 8–12. 1350074

[B62] Gosavi N. Lee H. J. Lee J. S. Patel S. Lee S. J. (2002). Golgi fragmentation occurs in the cells with prefibrillar alpha-synuclein aggregates and precedes the formation of fibrillar inclusion. J. Biol. Chem. 277, 48984–48992. 10.1074/jbc.M208194200 12351643

[B63] Graham D. G. (1978). Oxidative pathways for catecholamines in the genesis of neuromelanin and cytotoxic quinones. Mol. Pharmacol. 14, 633–643. 98706

[B64] Guardia-Laguarta C. Area-Gomez E. Rub C. Liu Y. Magrane J. Becker D.. (2014). alpha-Synuclein is localized to mitochondria-associated ER membranes. J. Neurosci. 34, 249–259. 10.1523/JNEUROSCI.2507-13.2014 24381286 PMC3866487

[B65] Hadjiconstantinou M. Neff N. H. (2008). Enhancing aromatic L-amino acid decarboxylase activity: implications for L-DOPA treatment in Parkinson's disease. CNS Neurosci. Ther. 14, 340–351. 10.1111/j.1755-5949.2008.00058.x 19040557 PMC6494005

[B66] Halbrugge T. Wolfel R. Graefe K. H. (1989). Plasma 3,4-dihydroxyphenylglycol as a tool to assess the role of neuronal uptake in the anaesthetized rabbit. Naunyn Schmiedebergs. Arch. Pharmacol. 340, 726–732. 10.1007/BF00169681 2634246

[B67] Hastings T. G. Berman S. B. (1999). Dopamine-induced toxicity and quinone modification of proteins: implications for Parkinson's disease, in Role of Catechol Quinone Species in Cellular Toxicity, ed C.R. Creveling (Johnson City, TN: F.P. Graham Publishing, Inc.), 69–89.

[B68] Herrera F. E. Chesi A. Paleologou K. E. Schmid A. Munoz A. Vendruscolo M.. (2008). Inhibition of alpha-synuclein fibrillization by dopamine is mediated by interactions with five C-terminal residues and with E83 in the NAC region. PLoS ONE 3:e3394. 10.1371/journal.pone.0003394 18852892 PMC2566601

[B69] Hetzenauer A. Sinnegger-Brauns M. J. Striessnig J. Singewald N. (2006). Brain activation pattern induced by stimulation of L-type Ca^2+^-channels: contribution of Ca(V)1.3 and Ca(V)1.2 isoforms. Neuroscience 139, 1005–1015. 10.1016/j.neuroscience.2006.01.059 16542784

[B70] Hirsch E. Graybiel A. M. Agid Y. A. (1988). Melanized dopaminergic neurons are differentially susceptible to degeneration in Parkinson's disease. Nature 334, 345–348. 10.1038/334345a0 2899295

[B71] Holford N. H. Chan P. L. Nutt J. G. Kieburtz K. Shoulson I. (2006). Disease progression and pharmacodynamics in Parkinson disease - evidence for functional protection with levodopa and other treatments. J. Pharmacokinet. Pharmacodyn. 33, 281–311. 10.1007/s10928-006-9012-6 16625427

[B72] Hopp S. C. Royer S. E. D'angelo H. M. Kaercher R. M. Fisher D. A. Wenk G. L. (2015). Differential neuroprotective and anti-inflammatory effects of L-type voltage dependent calcium channel and ryanodine receptor antagonists in the substantia nigra and locus coeruleus. J. Neuroimmune Pharmacol. 10, 35–44. 10.1007/s11481-014-9568-7 25318607 PMC4336597

[B73] Hurley M. J. Dexter D. T. (2012). Voltage-gated calcium channels and Parkinson's disease. Pharmacol. Ther. 133, 324–333. 10.1016/j.pharmthera.2011.11.006 22133841

[B74] Imaizumi T. Yamashita K. Taima K. Ishikawa A. Yoshida H. Satoh K. (2005). Effect of peroxisome proliferator-activated receptor-gamma ligands on the expression of retinoic acid-inducible gene-I in endothelial cells stimulated with lipopolysaccharide. Prostaglandins Other Lipid Mediat. 78, 46–54. 10.1016/j.prostaglandins.2005.02.006 16303604

[B75] Kalia L. V. Kalia S. K. Mclean P. J. Lozano A. M. Lang A. E. (2013). alpha-Synuclein oligomers and clinical implications for Parkinson disease. Ann. Neurol. 73, 155–169. 10.1002/ana.23746 23225525 PMC3608838

[B76] Kumer S. C. Vrana K. E. (1996). Intricate regulation of tyrosine hydroxylase activity and gene expression. J. Neurochem. 67, 443–462. 10.1046/j.1471-4159.1996.67020443.x 8764568

[B77] Lang A. E. Lozano A. M. (1998). Parkinson's disease. Second of two parts. N. Engl. J. Med. 339, 1130–1143. 10.1056/NEJM199810153391607 9770561

[B78] Larsen K. E. Schmitz Y. Troyer M. D. Mosharov E. Dietrich P. Quazi A. Z.. (2006). Alpha-synuclein overexpression in PC12 and chromaffin cells impairs catecholamine release by interfering with a late step in exocytosis. J. Neurosci. 26, 11915–11922. 10.1523/JNEUROSCI.3821-06.2006 17108165 PMC6674868

[B79] Lashuel H. A. Petre B. M. Wall J. Simon M. Nowak R. J. Walz T.. (2002). Alpha-synuclein, especially the Parkinson's disease-associated mutants, forms pore-like annular and tubular protofibrils. J. Mol. Biol. 322, 1089–1102. 10.1016/S0022-2836(02)00735-0 12367530

[B80] Lewitt P. A. (2015). Levodopa therapy for Parkinson's disease: pharmacokinetics and pharmacodynamics. Mov. Disord. 30, 64–72. 10.1002/mds.26082 25449210

[B81] Li H. T. Du H. N. Tang L. Hu J. Hu H. Y. (2002). Structural transformation and aggregation of human alpha-synuclein in trifluoroethanol: non-amyloid component sequence is essential and beta-sheet formation is prerequisite to aggregation. Biopolymers 64, 221–226. 10.1002/bip.10179 12115139

[B82] Lieberman O. J. Choi S. J. Kanter E. Saverchenko A. Frier M. D. Fiore G. M. (2017). Alpha-synuclein-dependent calcium entry underlies differential sensitivity of cultured, S. N., and VTA dopaminergic neurons to a Parkinsonian Neurotoxin. eNeuro 4:ENEURO.0167-17.2017. 10.1523/ENEURO.0167-17.2017 PMC5701296 29177188

[B83] Liu Y. Harding M. Pittman A. Dore J. Striessnig J. Rajadhyaksha A.. (2014). Cav1.2 and Cav1.3 L-type calcium channels regulate dopaminergic firing activity in the mouse ventral tegmental area. J. Neurophysiol. 112, 1119–1130. 10.1152/jn.00757.2013 24848473 PMC4122730

[B84] Lohr K. M. Bernstein A. I. Stout K. A. Dunn A. R. Lazo C. R. Alter S. P.. (2014). Increased vesicular monoamine transporter enhances dopamine release and opposes Parkinson disease-related neurodegeneration *in vivo*. Proc. Natl. Acad. Sci. U.S.A. 111, 9977–9982. 10.1073/pnas.1402134111 24979780 PMC4103325

[B85] Lotharius J. Brundin P. (2002). Impaired dopamine storage resulting from alpha-synuclein mutations may contribute to the pathogenesis of Parkinson's disease. Hum. Mol. Genet. 11, 2395–2407. 10.1093/hmg/11.20.2395 12351575

[B86] Lou H. Montoya S. E. Alerte T. N. Wang J. Wu J. Peng X.. (2010). Serine 129 phosphorylation reduces the ability of alpha-synuclein to regulate tyrosine hydroxylase and protein phosphatase 2A *in vitro* and *in vivo*. J. Biol. Chem. 285, 17648–17661. 10.1074/jbc.M110.100867 20356833 PMC2878529

[B87] Lowe R. Pountney D. L. Jensen P. H. Gai W. P. Voelcker N. H. (2004). Calcium(II) selectively induces alpha-synuclein annular oligomers via interaction with the C-terminal domain. Protein Sci. 13, 3245–3252. 10.1110/ps.04879704 15537754 PMC2287302

[B88] Malek N. Swallow D. Grosset K. A. Anichtchik O. Spillantini M. Grosset D. G. (2014). Alpha-synuclein in peripheral tissues and body fluids as a biomarker for Parkinson's disease - a systematic review. Acta Neurol. Scand. 130, 59–72. 10.1111/ane.12247 24702516

[B89] Marchitti S. A. Deitrich R. A. Vasiliou V. (2007). Neurotoxicity and metabolism of the catecholamine-derived 3,4-dihydroxyphenylacetaldehyde and 3,4-dihydroxyphenylglycolaldehyde: the role of aldehyde dehydrogenase. Pharmacol. Rev. 59, 125–150. 10.1124/pr.59.2.1 17379813

[B90] Martinez-Vicente M. Talloczy Z. Kaushik S. Massey A. C. Mazzulli J. Mosharov E. V.. (2008). Dopamine-modified alpha-synuclein blocks chaperone-mediated autophagy. J. Clin. Invest. 118, 777–788. 10.1172/JCI32806 18172548 PMC2157565

[B91] Masilamoni G. J. Bogenpohl J. W. Alagille D. Delevich K. Tamagnan G. Votaw J. R.. (2011). Metabotropic glutamate receptor 5 antagonist protects dopaminergic and noradrenergic neurons from degeneration in MPTP-treated monkeys. Brain 134, 2057–2073. 10.1093/brain/awr137 21705423 PMC3122374

[B92] Masliah E. Rockenstein E. Adame A. Alford M. Crews L. Hashimoto M.. (2005). Effects of alpha-synuclein immunization in a mouse model of Parkinson's disease. Neuron 46, 857–868. 10.1016/j.neuron.2005.05.010 15953415

[B93] Matschke L. A. Bertoune M. Roeper J. Snutch T. P. Oertel W. H. Rinne S.. (2015). A concerted action of L- and T-type Ca(2+) channels regulates locus coeruleus pacemaking. Mol. Cell. Neurosci. 68, 293–302. 10.1016/j.mcn.2015.08.012 26319746

[B94] Matsuda W. Furuta T. Nakamura K. C. Hioki H. Fujiyama F. Arai R.. (2009). Single nigrostriatal dopaminergic neurons form widely spread and highly dense axonal arborizations in the neostriatum. J. Neurosci. 29, 444–453. 10.1523/JNEUROSCI.4029-08.2009 19144844 PMC6664950

[B95] Mazzulli J. R. Armakola M. Dumoulin M. Parastatidis I. Ischiropoulos H. (2007). Cellular oligomerization of alpha-synuclein is determined by the interaction of oxidized catechols with a C-terminal sequence. J. Biol. Chem. 282, 31621–31630. 10.1074/jbc.M704737200 17785456

[B96] Mccaffery P. Drager U. C. (1994). High levels of a retinoic acid-generating dehydrogenase in the meso-telencephalic dopamine system. Proc. Natl. Acad. Sci. U.S.A. 91, 7772–7776. 10.1073/pnas.91.16.7772 8052659 PMC44484

[B97] Michel P. P. Hirsch E. C. Hunot S. (2016). Understanding dopaminergic cell death pathways in Parkinson disease. Neuron 90, 675–691. 10.1016/j.neuron.2016.03.038 27196972

[B98] Mironov S. L. (2015). alpha-Synuclein forms non-selective cation channels and stimulates ATP-sensitive potassium channels in hippocampal neurons. J. Physiol. 593, 145–159. 10.1113/jphysiol.2014.280974 25556793 PMC4293060

[B99] Mittal S. Bjornevik K. Im D. S. Flierl A. Dong X. Locascio J. J.. (2017). beta2-Adrenoreceptor is a regulator of the alpha-synuclein gene driving risk of Parkinson's disease. Science 357, 891–898. 10.1126/science.aaf3934 28860381 PMC5761666

[B100] Montine T. J. Farris D. B. Graham D. G. (1995). Covalent crosslinking of neurofilament proteins by oxidized catechols as a potential mechanism of Lewy body formation. J. Neuropathol. Exp. Neurol. 54, 311–319. 10.1097/00005072-199505000-00004 7745430

[B101] Mooslehner K. A. Chan P. M. Xu W. Liu L. Smadja C. Humby T.. (2001). Mice with very low expression of the vesicular monoamine transporter 2 gene survive into adulthood: potential mouse model for parkinsonism. Mol. Cell. Biol. 21, 5321–5331. 10.1128/MCB.21.16.5321-5331.2001 11463816 PMC87256

[B102] Mor D. E. Tsika E. Mazzulli J. R. Gould N. S. Kim H. Daniels M. J.. (2017). Dopamine induces soluble alpha-synuclein oligomers and nigrostriatal degeneration. Nat. Neurosci. 20, 1560–1568. 10.1038/nn.4641 28920936 PMC5893155

[B103] Mosharov E. V. Larsen K. E. Kanter E. Phillips K. A. Wilson K. Schmitz Y.. (2009). Interplay between cytosolic dopamine, calcium, and alpha-synuclein causes selective death of substantia nigra neurons. Neuron 62, 218–229. 10.1016/j.neuron.2009.01.033 19409267 PMC2677560

[B104] Mosharov E. V. Staal R. G. Bove J. Prou D. Hananiya A. Markov D.. (2006). Alpha-synuclein overexpression increases cytosolic catecholamine concentration. J. Neurosci. 26, 9304–9311. 10.1523/JNEUROSCI.0519-06.2006 16957086 PMC6674515

[B105] Mytilineou C. Han S. K. Cohen G. (1993). Toxic and protective effects of L-DOPA on mesencephalic cell cultures. J. Neurochem. 61, 1470–1478. 10.1111/j.1471-4159.1993.tb13642.x 8376999

[B106] Nakashima A. Kaneko Y. S. Mori K. Fujiwara K. Tsugu T. Suzuki T.. (2002). The mutation of two amino acid residues in the N-terminus of tyrosine hydroxylase (TH) dramatically enhances the catalytic activity in neuroendocrine AtT-20 cells. J. Neurochem. 82, 202–206. 10.1046/j.1471-4159.2002.00921.x 12091481

[B107] Naoi M. Dostert P. Yoshida M. Nagatsu T. (1993). N-methylated tetrahydroisoquinolines as dopaminergic neurotoxins. Adv. Neurol. 60, 212–217. 8420137

[B108] Nath S. Goodwin J. Engelborghs Y. Pountney D. L. (2011). Raised calcium promotes alpha-synuclein aggregate formation. Mol. Cell. Neurosci. 46, 516–526. 10.1016/j.mcn.2010.12.004 21145971

[B109] Nemani V. M. Lu W. Berge V. Nakamura K. Onoa B. Lee M. K.. (2010). Increased expression of alpha-synuclein reduces neurotransmitter release by inhibiting synaptic vesicle reclustering after endocytosis. Neuron 65, 66–79. 10.1016/j.neuron.2009.12.023 20152114 PMC3119527

[B110] Niethammer M. Feigin A. Eidelberg D. (2012). Functional neuroimaging in Parkinson's disease. Cold Spring Harb. Perspect. Med. 2:a009274. 10.1101/cshperspect.a009274 22553499 PMC3331691

[B111] Olanow C. W. (2015). Levodopa: effect on cell death and the natural history of Parkinson's disease. Mov. Disord. 30, 37–44. 10.1002/mds.26119 25502620

[B112] Olanow C. W. Agid Y. Mizuno Y. Albanese A. Bonuccelli U. Damier P.. (2004). Levodopa in the treatment of Parkinson's disease: current controversies. Mov. Disord. 19, 997–1005. 10.1002/mds.20243 15372588

[B113] Ottolini D. Cali T. Negro A. Brini M. (2013). The Parkinson disease-related protein DJ-1 counteracts mitochondrial impairment induced by the tumour suppressor protein p53 by enhancing endoplasmic reticulum-mitochondria tethering. Hum. Mol. Genet. 22, 2152–2168. 10.1093/hmg/ddt068 23418303

[B114] Ottolini D. Cali T. Szabo I. Brini M. (2017). Alpha-synuclein at the intracellular and the extracellular side: functional and dysfunctional implications. Biol. Chem. 398, 77–100. 10.1515/hsz-2016-0201 27508962

[B115] Outeiro T. F. Klucken J. Bercury K. Tetzlaff J. Putcha P. Oliveira L. M.. (2009). Dopamine-induced conformational changes in alpha-synuclein. PLoS ONE 4:e6906. 10.1371/journal.pone.0006906 19730729 PMC2731858

[B116] Pardo B. Mena M. A. Casarejos M. J. Paino C. L. De Yebenes J. G. (1995). Toxic effects of L-DOPA on mesencephalic cell cultures: protection with antioxidants. Brain Res. 682, 133–143. 10.1016/0006-8993(95)00341-M 7552304

[B117] Pasternak B. Svanstrom H. Nielsen N. M. Fugger L. Melbye M. Hviid A. (2012). Use of calcium channel blockers and Parkinson's disease. Am. J. Epidemiol. 175, 627–635. 10.1093/aje/kwr362 22387374

[B118] Peng X. Tehranian R. Dietrich P. Stefanis L. Perez R. G. (2005). Alpha-synuclein activation of protein phosphatase 2A reduces tyrosine hydroxylase phosphorylation in dopaminergic cells. J. Cell Sci. 118, 3523–3530. 10.1242/jcs.02481 16030137

[B119] Perez R. G. Waymire J. C. Lin E. Liu J. J. Guo F. Zigmond M. J. (2002). A role for alpha-synuclein in the regulation of dopamine biosynthesis. J. Neurosci. 22, 3090–3099. 11943812 10.1523/JNEUROSCI.22-08-03090.2002 PMC6757524

[B120] Perier C. Bove J. Vila M. (2012). Mitochondria and programmed cell death in Parkinson's disease: apoptosis and beyond. Antioxid. Redox Signal. 16, 883–895. 10.1089/ars.2011.4074 21619488

[B121] Pifl C. Rajput A. Reither H. Blesa J. Cavada C. Obeso J. A.. (2014). Is Parkinson's disease a vesicular dopamine storage disorder? Evidence from a study in isolated synaptic vesicles of human and nonhuman primate striatum. J. Neurosci. 34, 8210–8218. 10.1523/JNEUROSCI.5456-13.2014 24920625 PMC6608236

[B122] Rcom-H'cheo-Gauthier A. Goodwin J. Pountney D. L. (2014). Interactions between calcium and alpha-synuclein in neurodegeneration. Biomolecules 4, 795–811. 10.3390/biom4030795 25256602 PMC4192672

[B123] Richter D. (1937). Adrenaline and amine oxidase. Biochem. J. 31, 2022–2028. 10.1042/bj0312022 16746544 PMC1267175

[B124] Rochet J. C. Outeiro T. F. Conway K. A. Ding T. T. Volles M. J. Lashuel H. A.. (2004). Interactions among alpha-synuclein, dopamine, and biomembranes: some clues for understanding neurodegeneration in Parkinson's disease. J. Mol. Neurosci. 23, 23–34. 10.1385/JMN:23:1-2:023 15126689

[B125] Ronzitti G. Bucci G. Emanuele M. Leo D. Sotnikova T. D. Mus L. V.. (2014). Exogenous alpha-synuclein decreases raft partitioning of Cav2.2 channels inducing dopamine release. J. Neurosci. 34, 10603–10615. 10.1523/JNEUROSCI.0608-14.2014 25100594 PMC6802592

[B126] Sanchez-Padilla J. Guzman J. N. Ilijic E. Kondapalli J. Galtieri D. J. Yang B.. (2014). Mitochondrial oxidant stress in locus coeruleus is regulated by activity and nitric oxide synthase. Nat. Neurosci. 17, 832–840. 10.1038/nn.3717 24816140 PMC4131291

[B127] Schmitz Y. Lee C. J. Schmauss C. Gonon F. Sulzer D. (2001). Amphetamine distorts stimulation-dependent dopamine overflow: effects on D2 autoreceptors, transporters, and synaptic vesicle stores. J. Neurosci. 21, 5916–5924. 11487614 10.1523/JNEUROSCI.21-16-05916.2001 PMC6763160

[B128] Schnaitman C. Erwin V. G. Greenawalt J. W. (1967). The submitochondrial localization of monoamine oxidase. An enzymatic marker for the outer membrane of rat liver mitochondria. J. Cell. Biol. 32, 719–735. 10.1083/jcb.32.3.719 4291912 PMC2107278

[B129] Schneider J. L. Suh Y. Cuervo A. M. (2014). Deficient chaperone-mediated autophagy in liver leads to metabolic dysregulation. Cell Metab. 20, 417–432. 10.1016/j.cmet.2014.06.009 25043815 PMC4156578

[B130] Schneider J. L. Villarroya J. Diaz-Carretero A. Patel B. Urbanska A. M. Thi M. M.. (2015). Loss of hepatic chaperone-mediated autophagy accelerates proteostasis failure in aging. Aging Cell 14, 249–264. 10.1111/acel.12310 25620427 PMC4364837

[B131] Simon-Sanchez J. Schulte C. Bras J. M. Sharma M. Gibbs J. R. Berg D.. (2009). Genome-wide association study reveals genetic risk underlying Parkinson's disease. Nat. Genet. 41, 1308–1312. 10.1038/ng.487 19915575 PMC2787725

[B132] Specht C. G. Schoepfer R. (2001). Deletion of the alpha-synuclein locus in a subpopulation of C57BL/6J inbred mice. BMC Neurosci. 2:11. 10.1186/1471-2202-2-11 11591219 PMC57740

[B133] Spencer J. P. Whiteman M. Jenner P. Halliwell B. (2002). 5-s-Cysteinyl-conjugates of catecholamines induce cell damage, extensive DNA base modification and increases in caspase-3 activity in neurons. J. Neurochem. 81, 122–129. 10.1046/j.1471-4159.2002.00808.x 12067224

[B134] Spillantini M. G. Schmidt M. L. Lee V. M. Trojanowski J. Q. Jakes R. Goedert M. (1997). Alpha-synuclein in Lewy bodies. Nature 388, 839–840. 10.1038/42166 9278044

[B135] Stefanis L. (2012). Alpha-Synuclein in Parkinson's disease. Cold Spring Harb. Perspect. Med. 2:a009399. 10.1101/cshperspect.a009399 22355802 PMC3281589

[B136] Subramaniam M. Althof D. Gispert S. Schwenk J. Auburger G. Kulik A.. (2014). Mutant alpha-synuclein enhances firing frequencies in dopamine substantia nigra neurons by oxidative impairment of A-type potassium channels. J. Neurosci. 34, 13586–13599. 10.1523/JNEUROSCI.5069-13.2014 25297088 PMC6608377

[B137] Sulzer D. Bogulavsky J. Larsen K. E. Behr G. Karatekin E. Kleinman M. H. (2000). Neuromelanin biosynthesis is driven by excess cytosolic catecholamines not accumulated by synaptic vesicles. Proc. Natl. Acad. Sci. U.S.A. 97, 11869–11874. 10.1073/pnas.97.22.11869 11050221 PMC17261

[B138] Sulzer D. Zecca L. (2000). Intraneuronal dopamine-quinone synthesis: a review. Neurotox. Res. 1, 181–195. 10.1007/BF03033289 12835101

[B139] Surmeier D. J. Guzman J. N. Sanchez-Padilla J. Goldberg J. A. (2010). What causes the death of dopaminergic neurons in Parkinson's disease? Prog. Brain Res. 183, 59–77. 10.1016/S0079-6123(10)83004-3 20696315

[B140] Surmeier D. J. Obeso J. A. Halliday G. M. (2017a). Selective neuronal vulnerability in Parkinson disease. Nat. Rev. Neurosci. 18, 101–113. 10.1038/nrn.2016.178 28104909 PMC5564322

[B141] Surmeier D. J. Schumacker P. T. (2013). Calcium, bioenergetics, and neuronal vulnerability in Parkinson's disease. J. Biol. Chem. 288, 10736–10741. 10.1074/jbc.R112.410530 23086948 PMC3624453

[B142] Surmeier D. J. Schumacker P. T. Guzman J. D. Ilijic E. Yang B. Zampese E. (2017b). Calcium and Parkinson's disease. Biochem. Biophys. Res. Commun. 483, 1013–1019. 10.1016/j.bbrc.2016.08.168 27590583 PMC5449761

[B143] Swart T. Hurley M. J. (2016). Calcium channel antagonists as disease-modifying therapy for Parkinson's disease: therapeutic rationale and current status. CNS Drugs 30, 1127–1135. 10.1007/s40263-016-0393-9 27826740

[B144] Swirski M. Miners J. S. De Silva R. Lashley T. Ling H. Holton J.. (2014). Evaluating the relationship between amyloid-beta and alpha-synuclein phosphorylated at Ser129 in dementia with Lewy bodies and Parkinson's disease. Alzheimers Res. Ther. 6:77. 10.1186/s13195-014-0077-y 25452767 PMC4248436

[B145] Taylor T. N. Caudle W. M. Miller G. W. (2011). VMAT2-deficient mice display nigral and extranigral pathology and motor and nonmotor symptoms of Parkinson's disease. Parkinsons Dis. 2011:124165. 10.4061/2011/124165 21403896 PMC3043293

[B146] Tehranian R. Montoya S. E. Van Laar A. D. Hastings T. G. Perez R. G. (2006). Alpha-synuclein inhibits aromatic amino acid decarboxylase activity in dopaminergic cells. J. Neurochem. 99, 1188–1196. 10.1111/j.1471-4159.2006.04146.x 16981894

[B147] Tofaris G. K. Garcia Reitbock P. Humby T. Lambourne S. L. O'connell M. Ghetti B.. (2006). Pathological changes in dopaminergic nerve cells of the substantia nigra and olfactory bulb in mice transgenic for truncated human alpha-synuclein(1-120): implications for Lewy body disorders. J. Neurosci. 26, 3942–3950. 10.1523/JNEUROSCI.4965-05.2006 16611810 PMC6673887

[B148] Tyce G. M. Hunter L. W. Ward L. E. Rorie D. K. (1995). Effluxes of 3,4-dihydroxyphenylalanine, 3,4-dihydroxyphenylglycol, and norepinephrine from four blood vessels during basal conditions and during nerve stimulation. J. Neurochem. 64, 833–841. 10.1046/j.1471-4159.1995.64020833.x 7830077

[B149] Uhl G. R. (1998). Hypothesis: the role of dopaminergic transporters in selective vulnerability of cells in Parkinson's disease. Ann. Neurol. 43, 555–560. 10.1002/ana.410430503 9585349

[B150] Ulmer T. S. Bax A. Cole N. B. Nussbaum R. L. (2005). Structure and dynamics of micelle-bound human alpha-synuclein. J. Biol. Chem. 280, 9595–9603. 10.1074/jbc.M411805200 15615727

[B151] Van Dijk K. D. Berendse H. W. Drukarch B. Fratantoni S. A. Pham T. V. Piersma S. R.. (2012). The proteome of the locus ceruleus in Parkinson's disease: relevance to pathogenesis. Brain Pathol. 22, 485–498. 10.1111/j.1750-3639.2011.00540.x 21988495 PMC8057674

[B152] Vauzour D. Ravaioli G. Vafeiadou K. Rodriguez-Mateos A. Angeloni C. Spencer J. P. (2008). Peroxynitrite induced formation of the neurotoxins 5-S-cysteinyl-dopamine and DHBT-1: implications for Parkinson's disease and protection by polyphenols. Arch. Biochem. Biophys. 476, 145–151. 10.1016/j.abb.2008.03.011 18394421

[B153] Wakamatsu K. Tabuchi K. Ojika M. Zucca F. A. Zecca L. Ito S. (2015). Norepinephrine and its metabolites are involved in the synthesis of neuromelanin derived from the locus coeruleus. J. Neurochem. 135, 768–776. 10.1111/jnc.13237 26156066 PMC5014224

[B154] Werner-Allen J. W. Dumond J. F. Levine R. L. Bax A. (2016). Toxic dopamine metabolite DOPAL forms an unexpected dicatechol pyrrole adduct with lysines of alpha-Synuclein. Angew. Chem. Int. Ed Engl. 55, 7374–7378. 10.1002/anie.201600277 27158766 PMC4983193

[B155] Wey M. C. Fernandez E. Martinez P. A. Sullivan P. Goldstein D. S. Strong R. (2012). Neurodegeneration and motor dysfunction in mice lacking cytosolic and mitochondrial aldehyde dehydrogenases: implications for Parkinson's disease. PLoS ONE 7:e31522. 10.1371/journal.pone.0031522 22384032 PMC3284575

[B156] Xu J. Kao S. Y. Lee F. J. Song W. Jin L. W. Yankner B. A. (2002). Dopamine-dependent neurotoxicity of alpha-synuclein: a mechanism for selective neurodegeneration in Parkinson disease. Nat. Med. 8, 600–606. 10.1038/nm0602-600 12042811

[B157] Yamada T. Mcgeer P. L. Baimbridge K. G. Mcgeer E. G. (1990). Relative sparing in Parkinson's disease of substantia nigra dopamine neurons containing calbindin-D28K. Brain Res. 526, 303–307. 10.1016/0006-8993(90)91236-A 2257487

[B158] Zaichick S. V. Mcgrath K. M. Caraveo G. (2017). The role of Ca(2+) signaling in Parkinson's disease. Dis. Model. Mech. 10, 519–535. 10.1242/dmm.028738 28468938 PMC5451174

[B159] Zaltieri M. Longhena F. Pizzi M. Missale C. Spano P. Bellucci A. (2015). Mitochondrial dysfunction and alpha-synuclein synaptic pathology in Parkinson's disease: who's on first? Parkinsons Dis. 2015:108029. 10.1155/2015/108029 25918668 PMC4396726

[B160] Zhang F. Dryhurst G. (1994). Effects of L-cysteine on the oxidation chemistry of dopamine: new reaction pathways of potential relevance to idiopathic Parkinson's disease. J. Med. Chem. 37, 1084–1098. 10.1021/jm00034a006 7909337

[B161] Zhou Q. Yen A. Rymarczyk G. Asai H. Trengrove C. Aziz N.. (2016). Impairment of PARK14-dependent Ca(2+) signalling is a novel determinant of Parkinson's disease. Nat. Commun. 7:10332. 10.1038/ncomms10332 26755131 PMC4729940

[B162] Zucca F. A. Basso E. Cupaioli F. A. Ferrari E. Sulzer D. Casella L.. (2014). Neuromelanin of the human substantia nigra: an update. Neurotox. Res. 25, 13–23. 10.1007/s12640-013-9435-y 24155156

